# Perspectives: a journal on the cutting edge of educational research and development. Introduction from the new Editor in Chief

**DOI:** 10.1007/s40037-013-0105-9

**Published:** 2013-12-17

**Authors:** Erik Driessen

**Affiliations:** Maastricht, the Netherlands

As the new Editor in Chief, I will use this editorial to give a brief outline of my vision for the future of the journal. As the english-language successor to the dutch-language journal of the Dutch Association for Medical Education (NVMO), ‘Perspectives on Medical Education’ has become a fully-fledged international scientific journal. I commend the editorial board on this achievement. It provides me, together with the editorial board, with an excellent starting point for further growth. We opt to position the journal on the cutting edge of educational research and development, offering quality papers that are of interest to teachers, educational developers, policy makers and researchers: a position between more theory-oriented journals, such as Advances in Health Sciences Education and Medical Education on one hand and on the other hand journals with a more practical focus, such as clinical teacher. We invite authors to submit papers that focus on the transition of theory to practice and if possible on the impact on patient care. Clinicians are an important target group for Perspectives. In the coming period we will communicate this course through the editorials, commentaries, some new rubrics and manuscript categories. Firstly, we will update and globalize the Practical Guidelines to Clinical Education that were developed for the dutch-language version of the journal. Two associate editors—Bob McKinley and Fedde Scheele—will invite international experts to update the existing guidelines for practice on topics such as multi-source feedback, entrustable professional activities, and the interpretation of assessment data. Second, a new section for papers in which experienced experts give a state of the art overview of their field has been added. The first author to contribute to this section is one of our new editors—Cees van der Vleuten. In one of the coming issues he will give his perspective on how evidence can be translated to medical education. Third, we would like to focus the journal more broadly than medicine and also give space to the other health professions. We will make this more explicit in the definition of the aim of the journal and in the membership of the editorial board. In line with this is our plan for a section for papers on inter-professional education. Finally, we will start a section with papers that give a policy perspective to medical education. We will appoint associate editors with expertise in these domains.

In my opinion, it is essential that Perspectives achieves an impact factor, as this is likely to entice significantly more researchers to opt for Perspectives as the platform for their publications. Important requirements for eligibility for an impact factor are: papers which are cited by other researchers in the field, reliable and timely publication of the journal, short lead time of submitting an article to decision and possible publication, and an international team of editors. We will focus our attention on good quality papers and a timely response to authors. For good quality papers, constructive feedback from editors and reviewers to the authors is essential. To achieve this, the review process has been reorganized. The Editor in Chief reads and makes an initial assessment of all incoming papers. At this stage, unsuitable papers are rejected and others are sent for assessment to one of the associate editors—who are committed to the journal and have a network of potential reviewers. The associate editor rejects or sends the paper for review to a reviewer who is knowledgeable on the topic in question. These first two screenings by the Editor in Chief and associate editor will promote economical use of reviewer time without concessions as regards the quality of the review process.

## Changes in the editorial board

I would like to thank my predecessor Jan Borleffs for his contributions to the development of perspectives. Under his 12 years of leadership, the journal has developed into a truly international journal both in terms of authors and readers. Perspectives’ articles are downloaded by readers from all over the world, and each issue includes authors from different countries. For example, authors from Belgium, Canada, Ireland, New Zeeland, Saudi Arabia, the United Arab Emirates, UK, and USA contributed to this issue. In addition to Jan Borleffs, Onno Terpstra has also left the editorial board. I would like to thank Onno for all his work for the journal.

Fortunately, we have been able to expand the editorial board with new members. We are pleased to welcome the following persons:

- Bob McKinley of Keele University Medical School, Keele, Staffordshire, UK
- Brian Hodges of Toronto General Hospital, Toronto, ON, Canada
- Cees van der Vleuten of Maastricht University, Maastricht, the Netherlands
- Charlotte Ringsted of Toronto General Hospital, Toronto, ON, Canada
- Christopher Watling of Schulich School of Medicine and Dentistry, London, Canada
- Lorelei Lingard of Schulich School of Medicine and Dentistry, London, Canada
- Mahan Kulasegaram of Toronto General Hospital, Toronto, Toronto, ON, Canada
- Martin Grønnebæk Tolsgaard of Copenhagen University Hospital Rigshospitalet, Capital Region, Denmark.

We are very excited about the new members of the editorial board; they bring a new scope, energy and expertise to our team.

Our team is completed by Lieda Meester, editorial coordinator, Bohn Stafleu van Loghum.

With these new team members, we strive to make perspectives an exciting and thought-provoking journal that caters to the interests of all those involved in medical education.

